# First Principles Investigation of Binary Chromium Carbides Cr_7_C_3_, Cr_3_C_2_ and Cr_23_C_6_: Electronic Structures, Mechanical Properties and Thermodynamic Properties under Pressure

**DOI:** 10.3390/ma15020558

**Published:** 2022-01-12

**Authors:** Liang Sun, Xiongshuai Ji, Liang Zhao, Wenyan Zhai, Liujie Xu, Hui Dong, Yanmin Liu, Jianhong Peng

**Affiliations:** 1 National Joint Engineering Research Center for Abrasion Control and Molding of Metal Materials & Henan Key Laboratory of High-Temperature Structural and Functional Materials, Henan University of Science and Technology, Luoyang 471003, China; xlj@haust.edu.cn (L.X.); ymliu@xsyu.edu.cn (Y.L.); 2 Key Laboratory of Materials Processing Engineering, College of Materials Science and Engineering, Xi’an Shiyou University, Xi’an 710065, China; 19212050519@stumail.xsyu.edu.cn (X.J.); donghui@xsyu.edu.cn (H.D.); 3 Shaanxi Environmental Protection Group Ecological Construction Management Co., Ltd., Xi’an 710000, China; zeal228@163.com; 4 School of Physics and Electronic Information Engineering, Qinghai University for Nationalities, Xining 810007, China; pjh@qhmu.edu.cn

**Keywords:** binary chromium carbides (Cr_7_C_3_, Cr_3_C_2_, Cr_23_C_6_), first-principles, electronic structure, elastic properties, hardness anisotropy, debye temperature

## Abstract

Binary chromium carbides display excellent wear resistance, extreme stiffness and oxidation resistance under high temperature. The influence of applied pressure on electronic structure, elastic behavior, Debye temperature and hardness of Cr_7_C_3_, Cr_3_C_2_ and Cr_23_C_6_ have been investigated by the density functional theory (DFT) method. The results reveal that lattice parameters and formation enthalpy display an inverse relationship with applied pressure, and Cr_3_C_2_ exhibited optimal structural stability. Moreover, Cr-C orbital hybridization tends to be stronger due to the decreased partial density of states (PDOS) of the Cr atom. The difference in electronic distribution of binary carbides has also been investigated, which confirmed that overall orbital hybridization and covalent characteristics has been enhanced. The theoretical hardness was elevated according to the higher bond strength and bond density. In accordance with structural stability data, Cr_3_C_2_ has shown maximum theoretical hardness. Furthermore, the anisotropic nature of hardness has been evaluated with external pressure. Cr_3_C_2_, and the highest isotropic hardness behavior along with an increase in hardness values with increasing pressure has been observed. In addition, the variation in Debye temperatures of binary chromium carbides under applied pressure has also been predicted. The results provide a theoretical insight into electronic, mechanical and thermodynamic behavior of three binary chromium carbides and show the potential of these novel carbides in a wide range of applications.

## 1. Introduction

Chromium carbide ceramics have exhibited great promise in a wide range of applications, such as cutting tool industry, fabrication of surface electrodes, and wear-resistant coatings. These materials can also be used as grain refinement agents in cemented carbide and other wear-resistance components due to their high melting point, good wear resistance, extreme stiffness and outstanding oxidation resistance under a high temperature environment [1,2]. As shown in the balanced Cr-C binary phase diagram, three stable structures of binary chromium carbides [2,3,4,5,6,7,8,9,10,11], including Cr_7_C_3_ (Pnma), Cr_3_C_2_ (Pnma) and Cr_23_C_6_ (Fm-3m) can be found at ambient temperature and atmospheric pressure, which have been extensively researched by several groups [2,3,4,5,6,7,8,9,10,11,12,13,14,15,16,17,18]. 

Cr_3_C_2_ has already been widely studied and used for industrial applications, due to its excellent mechanical and thermodynamic properties [8,9,10,11,12,13,14,15]. For instance, Cintho et al. [5] have fabricated high-purity Cr_7_C_3_ and Cr_3_C_2_ powders by using a high-energy ball-mill, followed by heat treatment at 800 °C for two hours. Zhang et al. [14] have observed that the thicker layer of Cr_3_C_2_ in electronic packing results in fine particles and improves its mechanical properties. Hirota et al. [12], Esteve et al. [13] and Hussainova et al. [15] have studied the mechanical properties of Cr_7_C_3_, Cr_3_C_2_ and Cr_23_C_6_ phases and reported Vicker hardness (*H*_ν_) values in the range of *H*_ν_ (Cr_7_C_3_) = 5.71–17.0 GPa [12], 12.7–15.0 GPa [13], 21.0–22.0 GPa [15]. *H*_ν_ (Cr_3_C_2_) = 15.1–18.9 GPa [12], 20–22 GPa [13], 17.4–18.5 GPa [15]; *H*_ν_ (Cr_23_C_6_) = 12.7–15.0 GPa [12]. It is worth noting that the hardness values reported by different research groups are quite different due to the large variation in sample preparation methods and measurement protocols. Furthermore, several mechanical and electronic properties of these compounds cannot be experimentally measured due to certain experimental limitations, which highlights the significance and utilization of computational studies. On the theoretical front, Music et al. [7] have investigated the electronic structure and mechanical properties of orthogonal Cr_7_C_3_ based on first-principles and found that Cr-C bonds immerse in a large number of free electrons as the atomic chain of Cr-C-Cr, signifying the covalent bonding in orthogonal Cr_7_C_3_. Jiang et al. [9] have studied the phase structure, elastic behavior and electronic structure of different chromium carbides, and predicted the elastic modulus of these compounds for the first time. However, given the importance of high-pressure applications, such as aeronautics and astronautics, the performance of binary chromium carbides under pressure has not yet been explored. 

Herein, we have employed first-principles calculations on the lattice parameters, electronic structure, elastic modulus and thermodynamic properties of binary chromium carbides under pressure, ranging from 0 to 10 GPa. The changes in electronic structure and mechanical properties, such as theoretical hardness, hardness anisotropy, and thermodynamic properties of Cr_7_C_3_, Cr_3_C_2_ and Cr_23_C_6_ under external pressure have been predicted. The present study provides a theoretical base for future experimental research and exhibits the promise of chromium carbide ceramics in a wide range of applications. 

## 2. Calculation Methods and Models 

**Figure 1:**
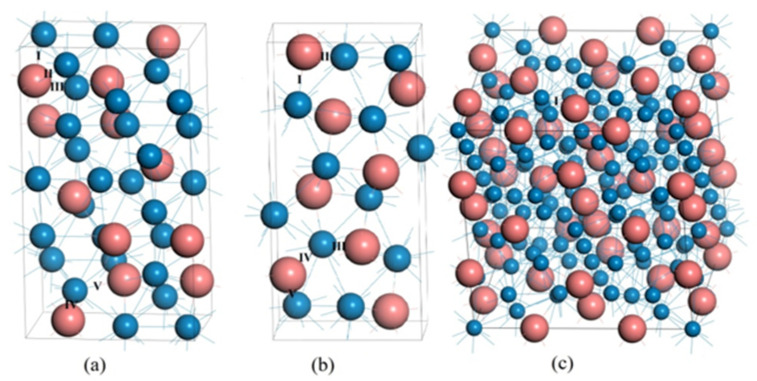
The crystal structure of (**a**) Cr_7_C_3_, (**b**) Cr_3_C_2_ and (**c**) Cr_23_C_6_.

All calculations on Cr_7_C_3_, Cr_3_C_2_ and Cr_23_C_6_ phases were performed by using the Cambridge Serial Total Energy Package (CASTEP) in Material Studio based on density functional theory (DFT) [19]. The Perdew-Burke-Ernzerhof (PBE) version of the generalized gradient approximation (GGA) was used to solve the equation of Kohn-Sham [20]. The ultra-soft pseudopotential method [21] was adopted to describe the interaction between valence electron and ion core [21], and the valence electrons for Cr and C atoms were 3*s*^2^ 3*p*^2^ 3*d*^5^ 4*s*^1^ and 2*s*^2^ 2*p*^2^, respectively. Based on the principle of minimum energy, all calculations were carried out in an inverted space and the cutoff energy of the plane wave was set to 350 eV. The number of *k*-points in the grid was in the Brillouin zone and *k*-points separation with the Monkhorst-Pack scheme for Cr_7_C_3_, Cr_3_C_2_ and Cr_23_C_6_ was 6 × 4 × 2, 6 × 10 × 2 and 2 × 2 × 2, respectively [22]. The Broyden-Fletcher-Goldfarb-Shannon (BFGS) algorithm was applied to relax the whole structure and attain a ground state where both cell parameters and fractional coordinates of atoms are optimized simultaneously. The conditions of geometric optimization are as follows: the convergence precision of total energy is 1 $\times \;10^{-6}$ eV/atom and the average force of each atom is within 0.002 eV/nm. The elastic constants were calculated by analyzing the changes in stress values resulting from the change in strain, i.e., stress–strain approach. The Parrinello-Rahman constant pressure method was used to change the pressure by adjusting the stress of primitive cell or primary cell, which affords shear force. The crystal structure of Cr_7_C_3_, Cr_3_C_2_ and Cr_23_C_6_ is shown in Figure 1. The pink-colored and blue-colored atoms represent Cr and C elements, respectively.

## 3. Results

### 3.1. Lattice Parameters

The optimized structural parameters of Cr_7_C_3_, Cr_3_C_2_ and Cr_23_C_6_ phases are given in Table 1. Moreover, previous experimental results and theoretical calculations are included for comparison. For all binary Cr-C phases, the calculated lattice constants are consistent with the previous experimental and theoretical studies, implying that the employed pseudopotentials are reliable.

**Figure 2:**
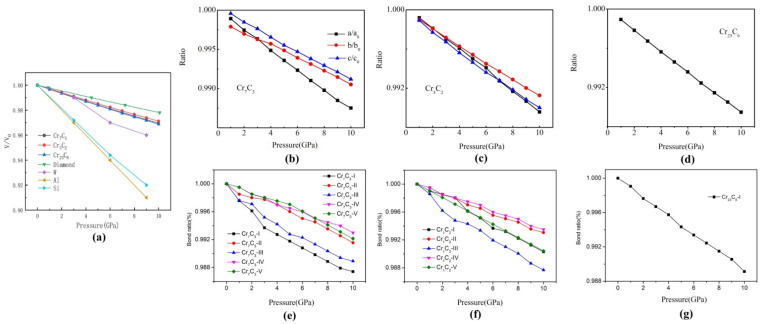
The chemical bonding properties of binary chromium carbides under hydrostatic pressure: (**a**) the unit cell volume rate (*V*/*V*_0_), (**b**) lattice parameters ratio (*a*/*a*_0_, *b*/*b*_0_, *c*/*c*_0_) of Cr_7_C_3_, (**c**) lattice parameters ratio (*a*/*a*_0_, *b*/*b*_0_, *c*/*c*_0_) of Cr_3_C_2_, (**d**) lattice parameters ratio (*a*/*a*_0_, *b*/*b*_0_, *c*/*c*_0_) of Cr_23_C_6_, (**e**) the chemical bonding properties of Cr_7_C_3_ under hydrostatic pressure, (**f**) the chemical bonding properties of Cr_3_C_2_ under hydrostatic pressure, (**g**) the chemical bonding properties of Cr_23_C_7_ under hydrostatic pressure [23].

The influence of applied pressure on the crystal structure of Cr_7_C_3_, Cr_3_C_2_ and Cr_23_C_6_ phases, the variation in the lattice parameters ratio (*a*/*a*_0_, *b*/*b*_0_, *c*/*c*_0_) and unit cell volume rate (*V*/*V*_0_) with respect to pressure are plotted in Figure 2a–d, where *a*_0_, *b*_0_, *c*_0_ and *V*_0_ are the zero-pressure equilibrium lattice parameters and volume.

It can be clearly observed that the ratio of *V/V*_0_ decreased along with increased pressure, which indicates that the crystal structure of each binary carbide undergoes a certain compression. Besides, authors compared the compressibility of W, Al, Si pure elements, which are always used as a matrix for chromium carbide composite. It can be seen that W metal displays closer compressibility trends with three binary carbides, while aluminum and silicon are much softer. At 10 GPa, the volume of Cr_7_C_3_, Cr_3_C_2_ and Cr_23_C_6_ was reduced by 3.05%, 2.89% and 3.12%, respectively, implying that Cr_3_C_2_ has optimal structural stability under applied pressure. The declining trend of *a*/*a*_0_ is obvious in the case of Cr_3_C_2_, which can be attributed to the longer Cr-C bond length along a-axis and the weaker bond strength. Similarly, the *c*/*c*_0_ of Cr_7_C_3_ shows a similar trend. 

The chemical bonding properties of binary chromium carbides under hydrostatic pressure are also discussed in Figure 2e–g. The chemical bonds, which are easy to change with pressure, are situated on the higher part of the graphs, showing a stronger dependence on pressure. The same type of bond V in Cr_7_C_3_ and bond IV in Cr_3_C_2_ are stiff and exhibit the smallest compressibility among all chemical bonds. Several different bond lengths are observed in binary chromium carbides and overall the variation trend remained. It can also be found that the bonds aligned along the (001) plane exhibit higher compressibility than other chemical bonds.

### 3.2. Formation Enthalpy

The formation enthalpy (Δ*H*) of binary chromium carbides has been calculated to evaluate the structural stability. The formation enthalpy is defined as the change in energy to form 1 M of any substance and a lower formation enthalpy indicates better forming ability. The formation enthalpy of a single cell is given below:

(1) $$△H=\frac{E_{total}({\mathrm{Cr}}_{x}C_{y})-xE_{bulk}-yE_{bulk}(C)}{x+y}$$

In Equation (1), *E_total_* (Cr*_x_*C*_y_*) represents the total cell energy; *E_bulk_*(Cr) and *E_bulk_*(C) refer to the chemical potential of Cr, C atoms in the bulk state; x and y correspond to the number of Cr, C atoms in each cell, respectively. Generally, the value of Δ*H* < 0 implies that binary chromium carbides are structural stable. It can be seen from Table 1 that the formation enthalpy of Cr_7_C_3_, Cr_3_C_2_ and Cr_23_C_6_ is −0.157, −0.183 and −0.122 eV·atom^−1^ without pressure, respectively. Therefore, the binary chromium carbides are structurally stable with a stability sequence of Cr_3_C_2_ > Cr_7_C_3_ > Cr_23_C_6._

**Figure 3:**
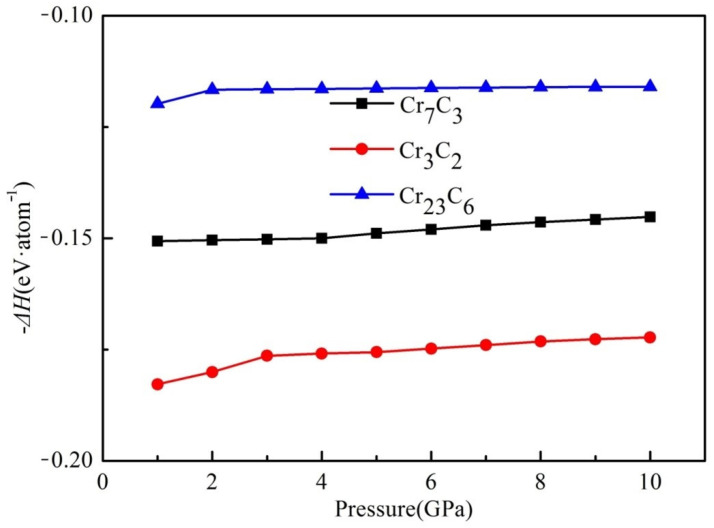
The formation enthalpy of binary chromium carbides under elevated pressure.

To investigate the structural stability of binary chromium carbides under pressure, the formation enthalpy of binary chromium carbides under elevated pressure ranging from 0 to 10 GPa is calculated and plotted in Figure 3. It can be seen that the formation enthalpy increased along with pressure, suggesting a lower structural stability in the binary phases. Furthermore, the initial growth (from 1 to 4 GPa) is more obvious than the latter (from 5 to 10 GPa), and the formation enthalpy of Cr_3_C_2_ kept on increasing, whereas the Δ*H* values for Cr_7_C_3_ and Cr_23_C_6_ remained at the pressure of 5–10 GPa.

Although the formation enthalpies of three compounds experienced a certain reduction, the negative values below 10 GPa indicate the structural stability of the three compounds. Moreover, Cr_3_C_2_ has exhibited the highest structural stability with a formation enthalpy of −0.172 eV·atom ^−1^ at 10 GPa.

### 3.3. Electronic Structures

The values of total density of states of binary chromium carbides at Fermi level N (*D*_f_), without any applied pressure, are shown in Table 1. The *D*_f_ values of 0.42, 0.36 and 0.41 state/(eV·atom) are observed for Cr_7_C_3_, Cr_3_C_2_ and Cr_23_C_6_, respectively. Moreover, the positive values of *D*_f_ indicates the metallic character of these compounds. Among them, the lower *D*_f_ value of Cr_3_C_2_ confirms that it has the weakest metallic behavior and highest structural stability, which is consistent with our formation enthalpy data.

**Figure 4:**
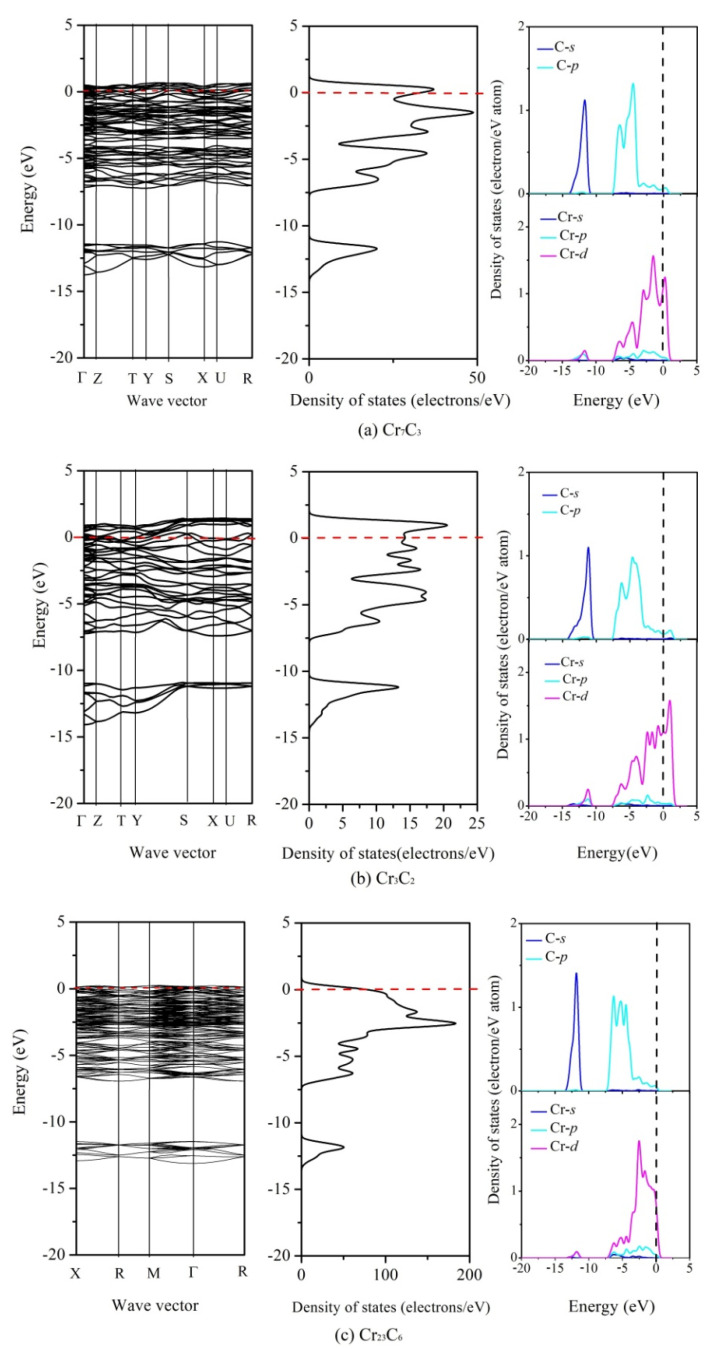
The total and partial density of states (DOS) of binary chromium carbides without applied pressure.

To further understand the basic features of the chemical bonding and phase stability, the total and partial density of states (DOS) of binary chromium carbides were calculated and shown in Figure 4. The main bonding peaks of binary chromium carbides, between −75 and −70 eV, are dominated by the valence electron number of the Cr (3*s*) orbit, while the main bonding peaks between −45 and −40 eV originated from the valence electron number of the Cr (3*p*) orbit. In addition, both of them have exhibited a strong local behavior. Meanwhile, the low energy region of three compounds between −14 and −10 eV is determined by the valence electron number of the C (2*s*) orbit, while among the high energy region, from −7 to −3 eV, this is contributed by Cr (3*d*) and C (2*p*) orbits, indicating that the covalent hybridization between the Cr and C atoms exits in these compounds. Moreover, the Cr (3*d*) predominates near the Fermi level stems because of its metallic characteristics. 

**Figure 5:**
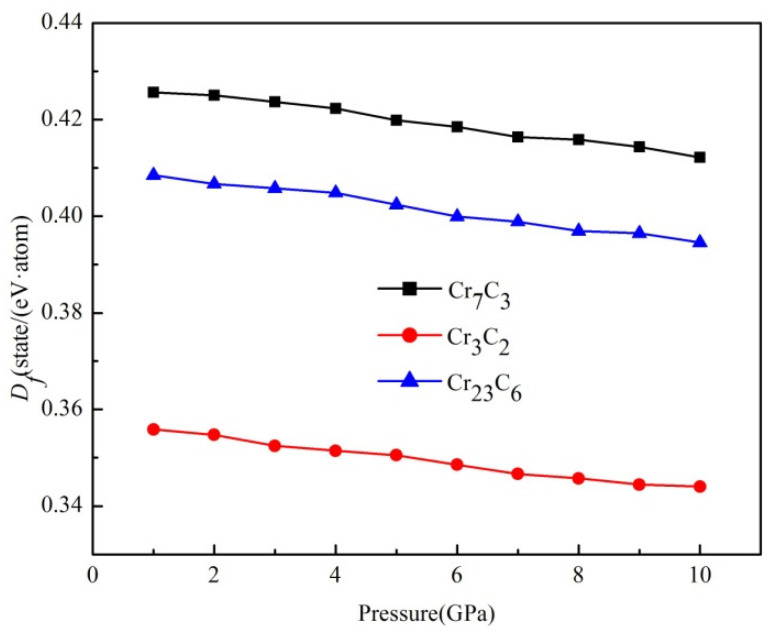
The value of the total DOS at Fermi level *N* (*D*_f_) decreases under pressure.

Figure 5 presents that the calculated value of the total DOS at Fermi level *N* (*D*_f_) decreases under pressure, which suggests that the metallic nature of Cr_7_C_3_, Cr_3_C_2_ and Cr_23_C_6_ reduced due to applied pressure. This probably attributes to the bond length of atoms, which becomes shorter under high pressure, and alters the interaction potentials. In addition, a pseudo-gap exists around the Fermi level and becomes wider under applied pressure. In general, the wider pseudo-gap represents a stronger covalent bond, which is in good agreement with our calculations.

**Figure 6:**
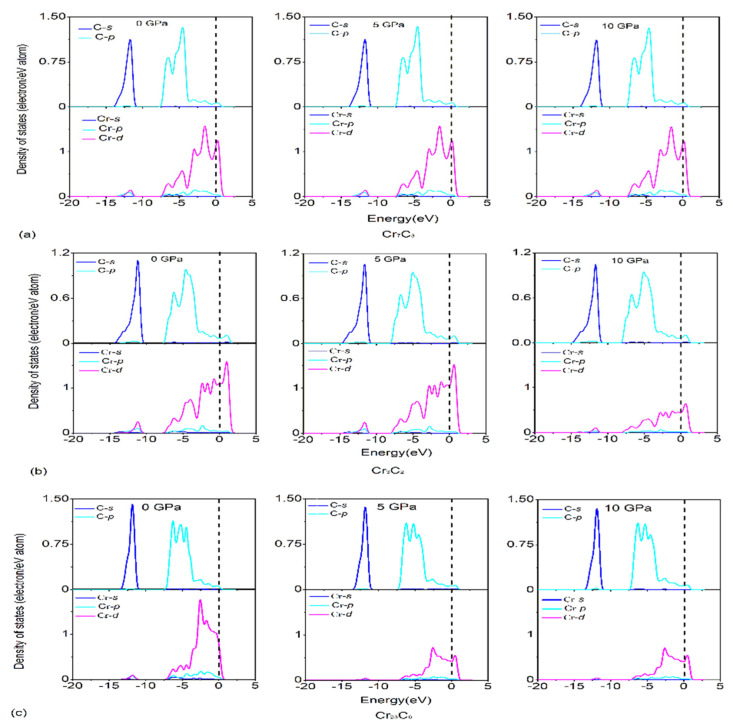
The partial DOS of (**a**) Cr_7_C_3_, (**b**) Cr_3_C_2_ and (**c**) Cr_23_C_6_ under pressure (0, 5 and 10 GPa).

Furthermore, the partial DOS under pressure (0, 5 and 10 GPa) is calculated and shown in Figure 6. It can be seen the partial DOS, especially for the Cr atom, decreased along with increasing pressure, which can be attributed to the reduced distance between atoms, wider pseudo-gaps, and higher bond strength of Cr-C.

**Figure 7:**
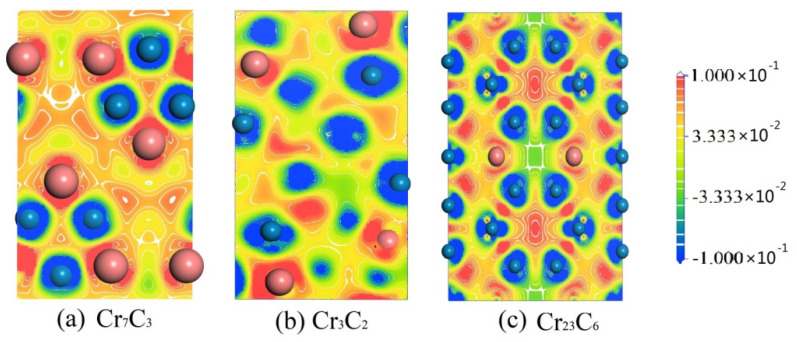
The charge density difference of three binary chromium carbides.

In order to visually demonstrate the covalent and ionic bonding characteristics, the charge density difference was measured and results are shown in Figure 7. The contour lines are plotted from −1 to 1 e/Å^3^. It can be clearly observed that the bonding type between C and Cr is covalent, the bonding between adjacent Cr atoms is metallic, whereas the bonding around C atoms is ionic. Based on the above results, it can also be seen that binary chromium carbides have strong structural stability.

**Figure 8:**
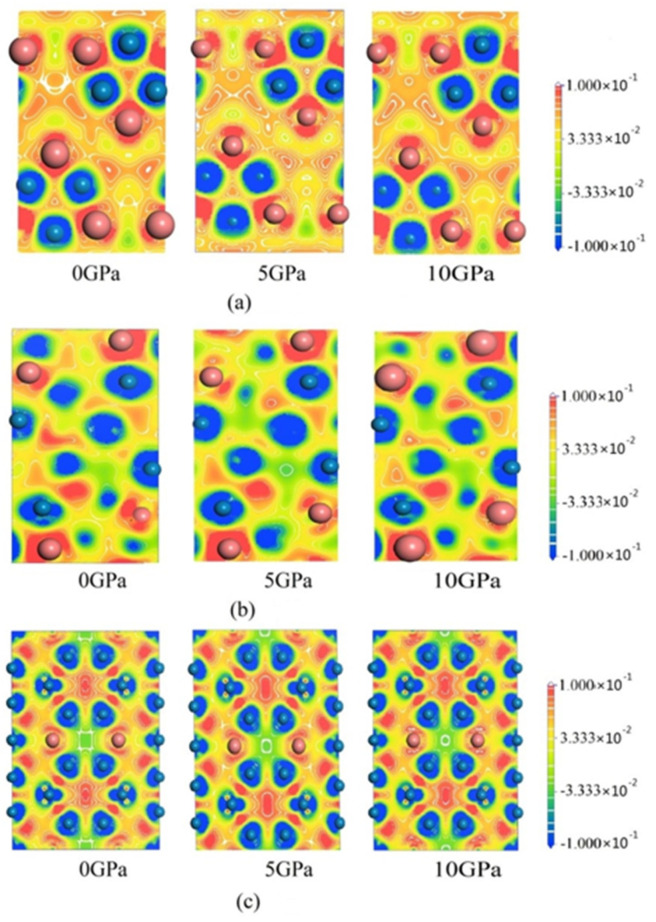
The charge density difference of (**a**) Cr_7_C_3_, (**b**) Cr_3_C_2_ and (**c**) Cr_23_C_6_ under different applied pressures.

Figure 8 shows the charge density difference of Cr_7_C_3_, Cr_3_C_2_ and Cr_23_C_6_ under different applied pressures (0, 5 and 10 GPa). It can be seen that the blue region surrounding Cr atoms decreased along with increasing pressure, which indicates that the energy difference between Cr and C atom shows a certain decrease. Moreover, it implies that the covalent bond becomes stronger due to the orbital hybridization. The color contrast of Cr_3_C_2_ clearly demonstrates that the covalent bond of Cr_3_C_2_ is the strongest among the chromium carbides.

### 3.4. Mechanical Characterizations

The mechanical properties of binary chromium carbides, such as elastic modulus and hardness are of critical importance in the wear resistance of surface coating materials. Moreover, most of the elastic properties remain almost in direct correlation with the elastic constants. In this work, the elastic constants of binary chromium carbides are calculated by the stress-strain approach and results are presented in Table 2. The mechanical stability criteria are expressed below [24,25,26,27,28,29,30]:

For orthorhombic system (Cr_7_C_3_, Cr_3_C_2_)

C_11_ + C_12_ + C_33_ + 2C_12_ + 2C_13_ + 2C_23_ > 0,

C_11_ + C_22_ > 2C_12_, C_22_ + C_33_ > 2C_23_,

C_11_ + C_33_ > 2C_13_, C_ii_ > 0 (I = 1–6)

For cubic system (Cr_23_C_6_):

C_11_ > 0, C_44_ > 0, C_11_ − C_12_ > 0, C_11_ + 2C_12_ > 0.

Table 2 confirms that chromium carbides have a mechanically stable structure because all binary Cr-C systems satisfy the stability criteria. Moreover, the results are consistent with other theoretical results. The mechanical properties, such as bulk modulus (*B*), shear modulus(*G*) and Young’s modulus (*E*), are obtained by using Voigt-Reuss-Hill (VRH) approximation, which considers the average of the bounds and provides the best estimation for mechanical properties of polycrystalline materials from known elastic constants of a single crystal [24]:

(2) $$B_{VRH}=\frac{1}{2}(B_{V}+B_{R})$$

(3) $$G_{VRH}=\frac{1}{2}(G_{V}+G_{R})$$

(4) $$E=\frac{9B_{VRH}G_{VRH}}{\left(3B_{VRH}+G_{VRH}\right)}$$

(5) $$v=\frac{\left(3B_{VRH}-2G_{VRH}\right)}{2(3B_{VRH}+G_{VRH})}$$

where *B_V_*, *B_R_* and *B_VRH_* represent the bulk modulus calculated by Voigt, Reuss and Voigt-Reuss-Hill approximation, respectively. Similarly, *G_v_*, *G_R_* and *G_VRH_* correspond to the shear modulus calculated from Voigt, Reuss and Voigt-Reuss-Hill approximation, respectively. *E* refers to the Young’s modulus and ν represents the Poisson’s ratio. The values of *B*, *G*, *E* and ν for Cr_7_C_3_, Cr_3_C_2_ and Cr_23_C_6_ at zero pressure are presented in Table 2, and are in good agreement with other theoretical results. *B* represents the resistance to fracture and Cr_7_C_3_, Cr_3_C_2_ and Cr_23_C_6_ resulted in *B* values of 314.7, 341.7 and 296.1 GPa, respectively. *G* represents the resistance to plastic deformation and Cr_3_C_2_ exhibited the maximum value of *G*. Furthermore, the *B*/*G* ratio is generally used to assess the ductile or brittle nature of materials. If *B*/*G* value is less than 1.75, the materials exhibit brittle behavior; whereas for *B*/*G* > 1.75, the material demonstrate ductile nature. *E* refers to the resistance to compression or tensile resistance, and Cr_7_C_3_, Cr_3_C_2_ and Cr_23_C_6_ have delivered E values of 353.4, 423.7 and 377.9 GPa, respectively. It is worth noting that these *E* values are higher than 3Al_2_O_3_-SiO_2_ (145 GPa) [34,35], ZrO_2_(160–241 GPa) [35], MgAl_2_O_4_(240 GPa) [35], Si_3_N_4_ (220–320 GPa) [36], and AlN(310–350 GPa) [36], and lower than TiC(379 GPa) [36], WC(400–650 GPa) [36], WC-Co(400–530 GPa) [36], diamond(1000 GPa) [36,37,38]. In brief, the Cr_3_C_2_ compound has demonstrated the highest values of *B*, *G* and *E*, which indicate that Cr_3_C_2_ has the greatest resistance to deformation.

**Figure 9:**
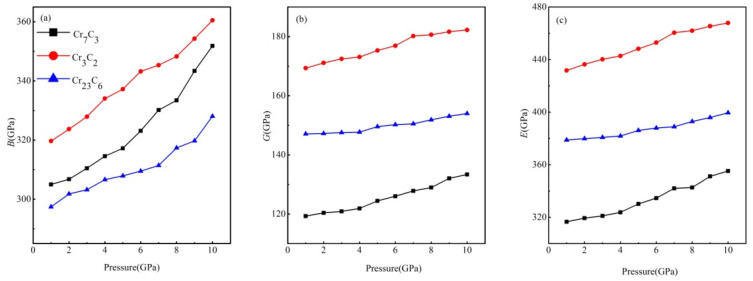
The values of (**a**) *B*, (**b**) *G* and (**c**) *E* of Cr_7_C_3_, Cr_3_C_2_ and Cr_23_C_6_ under applied pressure.

In addition, the values of *B*, *G* and *E* of Cr_7_C_3_, Cr_3_C_2_ and Cr_23_C_6_ under applied pressure are also calculated and presented in Figure 9. The calculated moduli have shown a certain similarity and a direct relationship with increase with pressure. In terms of *B*, the initial rate of growth, from 0 to 4 GPa, is less steep than the latter part, from 5 to 10 GPa. Moreover, the resistance to pressure becomes higher along with the elevated pressure. Furthermore, the values of *G* are lower than those of *B* from 0 to 10 GPa, which implies that the shear modulus limits the structural stability. In case of Young’s modulus, the growth rate for Cr_3_C_2_ was steeper than for Cr_7_C_3_ and Cr_23_C_6_, which can be attributed to the larger volumetric variations of Cr_3_C_2_ under applied pressure (Figure 2).

Meanwhile, hardness is an important index to assess wear resistance of ceramic materials. The hardness of a ceramic material has a certain relationship with its composition and microstructure. According to Gao’s theory [27], the theoretical hardness can be calculated by using the given empirical formulae:

(6) $$H={\left[\prod_{}^{u}(H_{v}^{u}\right)}^{n^{u}}{]}^{1/\sum n^{u}}$$

(7) $$H_{v}^{u}(GPa)=740P^{u}{\left(v_{b}^{u}\right)}^{-(5/3)}$$

(8) $$v_{b}^{u}=\frac{{\left(d^{u}\right)}^{3}}{\sum_{v}\left[{\left(d^{v}\right)}^{3}N_{b}^{v}\right]}=\frac{{\left(d^{u}\right)}^{3}}{\sum_{v}\left[{\left(d^{v}\right)}^{3}\frac{N^{v}}{\Omega }\right]}=\frac{{\left(d^{u}\right)}^{3}\Omega }{\sum_{v}\left[{\left(d^{v}\right)}^{3}N^{v}\right]}$$

where *H* refers to the theoretical hardness of compound, $H_{v}^{u}$ represents the hardness of *u* type bond, *d^u^* corresponds to the bond length, $H_{v}^{u}$ refers to the *v* type bond density per cubic angstroms; *N^v^* represents the total number of *v* type bonds in the cell, and *P^u^* and Ω correspond to the overlap population of *u* type bond and cell volume, respectively.

The overlap population of each atom is shown in Table 3. The difference of atomic position in three binary Cr-C systems resulted in few gaps in the gains and losses of Cr and C atoms. Total refers to the total electron number after the gains and losses; ***Charge*** refers to the gains and losses in electron number, where negative value indicates the electron gains and vice versa, and summation of both values provides the original electron number, such as 4.53 + (−0.53) = 4. It is well-known that the bigger overlap population of electron clouds implies the better capacity of a covalent bond, which confirms that Cr_3_C_2_ has the strongest covalent bond.

The calculated hardness of each Cr-C bond and binary chromium carbide, without external pressure, is shown in Table 4. The theoretical hardness of Cr_7_C_3_, Cr_3_C_2_ and Cr_23_C_6_ was found to be 13.5, 18.2 and 10.1 GPa, respectively. The difference in hardness stems from the difference in Cr-C bond strength and bond density. In spite of the lower bond density, the Cr_3_C_2_ compound has exhibited the highest hardness value and maximum Cr-C bond strength. The calculated results are in good agreement with the experimental studies [15]. However, the hardness values are lower than the values calculated by Min Ting et al. [18], where the semi-empirical formula of Šimůnek has been used to the calculate the hardness values [25]. This might have happened due to the computational difference in cell volume and Cr-C bond strength. However, it is worth emphasizing that our samples have shown a similar sequence for three compounds: Cr_3_C_2_ > Cr_7_C_3_ > Cr_23_C_6_. This implies that our results are acceptable based on a different calculation method.

Moreover, most of the carbide ceramics exhibit high hardness values, such as FeC (8.4 GPa) [10], TaN (11.0 GPa) [10] and NbN (13.3 GPa) [10], Mo_2_C (15.5 GPa) [10], TaC (16.7 GPa) [10] and NbC (19.6 GPa) [10], CrB_2_ (20.5 GPa [10], 23.0 GPa) [10] and VC (27.2 GPa) [10], α-B_4_C_3_ (60 GPa) [13] and *c*-B_4_C_3_ (65 GPa) [13]. One should note that the hardness values, achieved for binary chromium carbides, are reasonably high and located in the middle of this spectrum.

**Figure 10:**
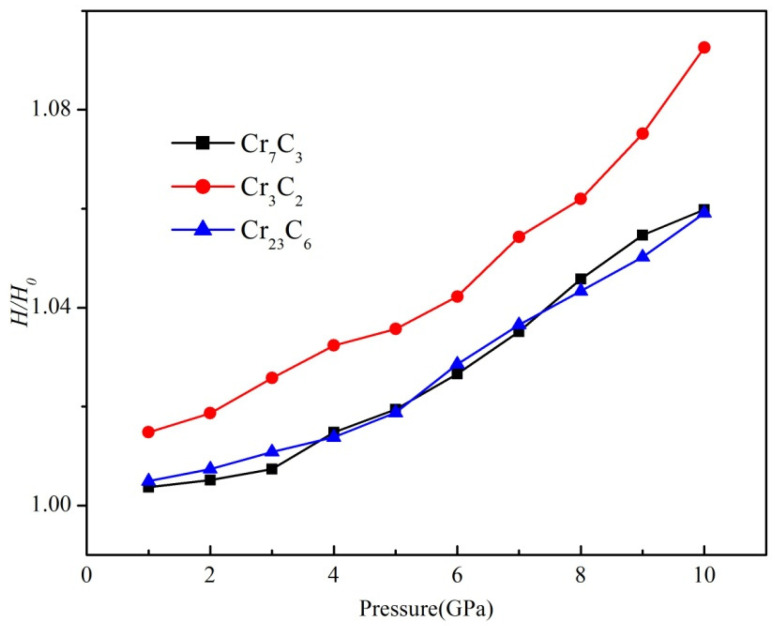
The relationship between *H/H*_0_ and applied pressure.

Furthermore, the materials should deliver high hardness under high-pressure environments. Hence, the hardness values under applied pressure are calculated, and the *H/H*_0_ ratio is used to characterize the change in hardness, where *H*_0_ refers to the hardness without external pressure. Figure 10 presents the relationship between *H/H*_0_ and applied pressure. It can be clearly observed that hardness values linearly increased with applied pressure, which can be attributed to the increase in bond density and shortened bond length. Moreover, the variation in *H/H*_0_ of Cr_3_C_2_ is more obvious than the Cr_7_C_3_ and Cr_23_C_6_, which shows that the influence of Cr-C bond strength and the density of Cr_3_C_2_ play a remarkable role. Cr_3_C_2_ has exhibited the highest strength under applied pressure. These calculations provide the theoretical basis for the development and utilization of chromium carbides, particularly Cr_3_C_2_, for a wide range of applications.

Hardness anisotropy also provides guidelines to fabricate the hardest cutting tools. Generally, machine components are desirable with cutting angle accuracy and specific shape. The hardest facets are designed to interface and orient with the machine component, which substantially enhances the service life of cutting tools. According to the Li’ theory, anisotropic hardness can be computed from the given equations [37,38,39]:

(9) $$Hani={\left[\prod_{i=1}^{j}H_{i}^{ni}\right]}^{1/\sum_{i=1}^{j}ni}$$

(10) $$Hi=a\delta_{t(i)}^{b}\rho_{i}^{c}$$

(11) $$\rho_{i}=n_{i}/V_{i}$$

(12) $$V_{i}=n_{i}d_{i}^{3}\;\;V/\sum_{k=1}^{j}n_{kd_{k}^{3}}$$

where *i* represents the *i*th department, *n_i_* refers to the bond number, ${\delta t}^{i}$ represents the total band strength, *ρ_i_* corresponds to the bond density, *V_i_* represents the volume, and *d_i_* refers to the bond length. The constants *a*, *b*, *c* take the value of 1.5, 1 and 0.5 for Vickers hardness, and 1.3, 1 and 0.5 for Knoop hardness. *δ_t_* can be obtained from the given equation [39]:

(13) $$\delta_{t}=\frac{\delta_{s}\delta_{b}}{{\delta_{s}\sin^{2}\varphi +\delta }_{b}\cos^{2}\varphi }$$

where *φ* refers to the angle between a specific bond and designated crystallographic direction or crystallographic plan, and the average values of sin*φ* and cos*φ* are π⁄4 and 1/2, respectively. *δ_s_* and *δ_b_* correspond to the stretching strength and bending strength, respectively, and can be obtained from the given relationships [39]:

(14) $$\delta_{s}=26.9\frac{{\chi_{A}\chi }_{B}}{d^{0.5}}$$

(15) $$\delta_{b}=33.5\frac{{\chi_{A}\chi }_{B}}{d^{2}}e^{-9.7fw}$$

where *z* represents the charge transfer number between bonded atoms A and B. The calculated anisotropic hardness values of binary chromium carbides are presented in Table 5. Overall, Cr_7_C_3_ exhibited the highest hardness due to shorter bond length and maximum bond density. For Cr7C3, the hardness in [110] direction is higher than other directions. Moreover, the hardness in [100] direction is higher than [010] and [001] directions, which are perpendicular to the a-axis. This phenomenon can be attributed to the bond angle. For example, the shortest bond of Cr7C3 is the Cr-C bond, which has a bond length of 1.974 Å. The Cr-C bond makes an angle of 23.391°, 69.479° and 87.38° with [100], [010] and [001] directions, respectively. It is worth noting that the smaller angle results in stronger δt which consequently results in higher hardness. Moreover, the hardness along [110] and [111] directions are similar to the average hardness, which is probably due to higher Cr-C covalent bonds in these directions as compared to the {110} family. In addition, the maximum covalent bonds are located in (110) plane. For Cr3C2, the hardness values along the given directions are consistent with the average hardness, which indicates that Cr-C bonds are homogeneously distributed. Hence, owing to excellent structural stability and mechanical performance, Cr3C2 has potential to be used in a wide range of applications, such as cutting tools and wear-resistant coatings. For Cr23C6, hardness remarkably dispersed along given directions, and the hardness value along [001] matches the overall hardness, though the average calculated hardness is higher than the previously published reports [12,13,14,15,40,41,42,43,44,45].These data are the first to provide prediction of hardness anisotropy for binary chromium carbides.

*χ_A_* and *χ_B_* represent the electronegativity of atoms of *A* and *B*, respectively, and *f_w_* refers to the strength weakening factor, which can be expressed as:

(16) $$f_{w}=\frac{\left|\chi_{A}-\chi_{B}\right|}{z^{2}\left(\chi_{A}-\chi_{B}\right)}$$

**Figure 11:**
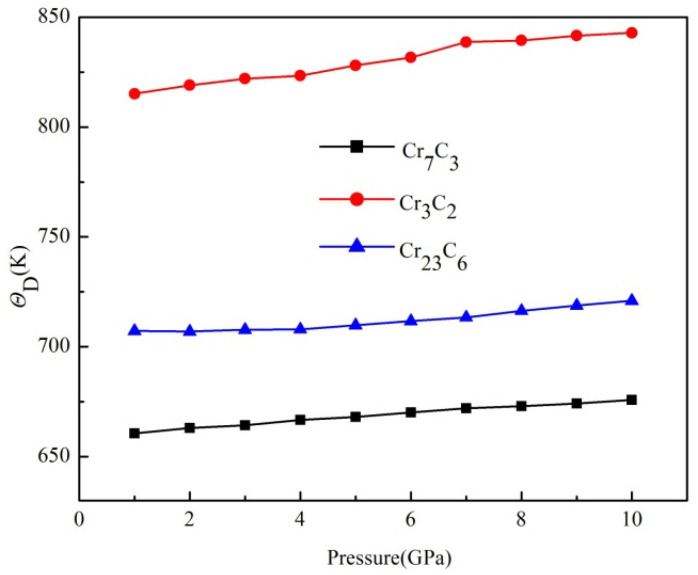
The variation in anisotropic hardness with respect to applied pressure.

To investigate the variation in anisotropic hardness with respect to applied pressure, the hardness values were calculated in a different direction, under the pressure of 1–10 GPa, and results are presented in Figure 11. Overall, the hardness values have shown a direct relationship with applied pressure. For Cr7C3, the increase in hardness value along [001] is most significant, whereas the minimum hardness value was observed along [001] direction at 0 GPa. This can be attributed to a relatively higher initial bond length, which becomes noticeably shorter with the reduction in crystal trend. Moreover, the hardness value along [010] direction in Cr3C2 and Cr23C6 has shown a similar condition. However, some differences have also been observed among three compounds. For instance, the rate of hardness increases along [110] was higher and steeper than the rate along [010] and [001] directions for Cr7C3, in the pressure range of 1–3 GPa. However, it becomes normal in the pressure range of 3–10 GPa, which is mainly due to the bond length along the [110] direction. The higher rate results in reduced amplitude of the inherent short bond, smaller than those bonds. For Cr_3_C_2_ and Cr_23_C_6_, the ratio curves exhibited a constant trend. In summary, the hardness of Cr_3_C_2_ has a relatively balanced increase compared to Cr_7_C_3_ and hardness value of Cr_3_C_2_ are higher than for Cr_23_C_6_, which confirms the desirable mechanical properties of Cr_3_C_2_.

### 3.5. Thermodynamic Stability

Debye temperature (Θ_*D*_) is the critical temperature applied in the energy equalization theorem. According to the Debye Law of solid physics, the specific heat tends to become 0 at extremely low temperatures and energy equalization is achieved if *T* > Θ_*D*_ [17]. To analyze the thermodynamic stability of binary chromium carbides, the Debye temperatures (Θ_*D*_) are calculated in this section. The Debye temperature has a certain correlation with many physical properties, such as melting point, elastic modulus and specific heat. Generally, the average sound velocity (*v_m_*) is used to calculate the Debye temperature, according to the given equation [46,47,48,49]:

(17) $$\Theta_{D}=\frac{h}{k_{B}}{\left[\frac{3n}{4\pi }\left(\frac{\rho N_{A}}{M}\right)\right]}^{\frac{1}{3}}v_{m}$$

where *h* refers to the Planck’s constant; *k_B_* represents the Boltzmann constant; *N_A_* corresponds to the Avogadro constant; *n* refers to the total number of atoms per formula; *ρ* represents the density; and *M* is the relative molecular mass per compound. $v_{m}$ can be calculated from the following expression:

(18) $$v_{m}={\left[\frac{1}{3}\left(\frac{2}{v_{s}^{3}}+\frac{1}{v_{1}^{3}}\right)\right]}^{-\frac{1}{3}}$$

where *v*_1_ and *v*_s_ represent the longitudinal and transverse sound velocities, respectively, which are calculated from the Navier’s formulas [50]:

(19) $$v_{1}=\sqrt{(B+\frac{4}{3}G)\frac{1}{\rho }},\;v_{s}=\sqrt{G/\rho }$$

The calculated results of Θ*_D_*, $v_{1}$. and $v_{s}$ are listed in Table 6. The Θ*_D_* is used to characterize the strength of covalent bonds in solids. It can be observed that the higher Θ*_D_* of 850 K was achieved for Cr_3_C_2_, which confirms the presence of strong covalent bonds in Cr_3_C_2_. This observation is consistent with our mechanical characterization data, where Cr_3_C_2_ has shown higher mechanical properties as compared to Cr_7_C_3_ and Cr_23_C_6_. In addition, the higher Debye temperature implies that thermal stability of Cr_3_C_2_ is higher than the other two compounds.

**Figure 12:**
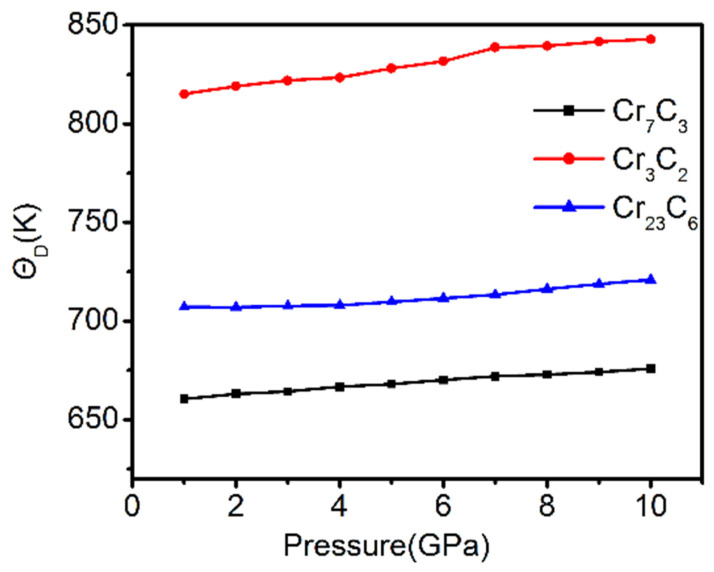
*Θ_D_* of binary chromium carbides under external pressure.

To study the influence of applied pressure on the thermodynamic stability of the binary chromium carbides, Θ*_D_* of binary chromium carbides, under external pressure, is calculated and presented in Figure 12. It can be found Θ*_D_* gradually increased along with increasing pressure, which implies the higher thermodynamic stability of Cr_7_C_3_, Cr_3_C_2_ and Cr_23_C_6_. The Θ*_D_* curves have shown a constant trend and the growth curve of Cr_3_C_2_ was relatively definite, which suggests that the thermodynamic stability of Cr_3_C_2_ is better than other two compounds. This result provides an insight into the highest bulk modulus of Cr_3_C_2_, which is correlated with the resistance to volumetric deformations. In brief, the Cr_3_C_2_ has exhibited the best mechanical and thermodynamic properties among the three binary Cr-C systems.

## 4. Conclusions

Herein, we have carried out an ab initio study of binary chromium carbides, under applied pressure, by using first-principles based on density functional theory and evaluated the crystal structure, electronic structure, mechanical performance and thermal properties. We have found that the *V/V*_0_ ratio decreased along with increasing pressure, and the axial position, with longer bond length, is easier to compress. In terms of formation enthalpy, Cr_7_C_3_, Cr_3_C_2_ and Cr_23_C_6_ are structurally stable under applied pressure ranging from 0 to 10 GPa, and Cr_3_C_2_ has exhibited the best structural stability (minimal formation enthalpy). The electronic structure investigations revealed that the chemical bonds in Cr_7_C_3_, Cr_3_C_2_ and Cr_23_C_6_ are of a mixed type: stronger covalent bonds, ionic bonds and weaker metallic bonds. Moreover, the largest pseudo-gaps and higher bond strength were observed with increasing pressure. Cr_3_C_2_ has shown the strongest covalent bond. The values of moduli, *B*, *G* and *E* of Cr_7_C_3_, Cr_3_C_2_ and Cr_23_C_6_ under applied pressure increased, which imply that crystal structures are difficult to alter under elevated pressure. The hardness values of Cr_7_C_3_, Cr_3_C_2_ and Cr_23_C_6_ are 13.5, 19.2 and 10.1 GPa without any external pressure (0 GPa), and increased with increasing pressure. Similar to electronic and structural properties, the Cr_3_C_2_ has exhibited the best mechanical properties. Furthermore, we have studied the hardness anisotropy and concluded that the hardness values in [110], [111] and [001] are highest for Cr_7_C_3_, Cr_3_C_2_ and Cr_23_C_6_, respectively. In addition, the Cr-C bonds of Cr_3_C_2_ were homogenously distributed and hardness anisotropy was not obvious. The initial minimum hardness increased rapidly under the influence of external pressure, which is probably due to the contraction of Cr-C bonds around these directions. Finally, the Debye temperature (Θ*_D_*) of Cr_7_C_3_, Cr_3_C_2_ and Cr_23_C_6_ are calculated and it has shown a direct relationship with pressure. These results confirm that binary compounds are thermodynamically stable.

## Figures and Tables

**Table 1:** Calculated parameters, formation enthalpy (Δ*H*), and DOS at the Fermi lever *D*_f_ of Cr_7_C_3_, Cr_3_C_2_ and Cr_23_C_6_ at zero pressure.

Compounds	Lattice Parameters (Å)			−2ΔH/ (eV·Atom)^−1^	*D*_f_/ State (eV·Atom)^−1^
	*a*	*b*	*c*		
Cr_7_C_3_	4.45 (4.51 *^a^*, 4.53 *^c^*)	6.84 (6.90 *^a^*, 7.01 *^c^*)	11.97 (12.08 ^*a*^, 12.14 *^c^*)	0.183 (0.112 ^*a*^, 0.149 *^b^*)	0.42 (0.43 *^a^*)
Cr_3_C_2_	5.48 (5.49 *^a^*, 5.54 *^d^*)	2.79 (2.79 *^a^*, 2.83 *^d^*)	11.47 (11.47 *^a^*, 11.49 *^d^*)	0.157 (0.15 *^b^*, 0.114 *^a^)*	0.36 (0.36 *^a^*)
Cr_23_C_6_	10.55 (10.55 *^a^*, 10.66 *^e^*)	10.55 (10.55 *^a^*, 10.66 *^e^*)	10.55 (10.55 *^a^*, 10.66 *^e^*)	0.122 (0.123 *^b^*, 0.087 *^a^*)	0.41 (0.42 *^a^*)

*^a^* Cal. data from Min et al. [18]. *^b^* Exp. Lattice parameters from [8]. *^c^* Exp. Lattice parameters from Ref [20]. *^d^* Exp. Lattice parameters from Ref [21]. *^e^* Exp. Lattice parameters from Ref [22].

**Table 2:** Calculated values of the independent elastic constants (C*_ij_*), C_11_/C_33_, C_12_/ C_13_, C_44_/ C_66_, bulk module *(B*, GPa), shear module (*G*, GPa), Young module (*E*, GPa), and Poisson’s ratio (*v*) at zero pressure.

Phase	C_11_/GPa	C_12_/GPa	C_13_/GPa	C_22_/GPa	C_23_/GPa	C_33_/GPa	C_44_/GPa	C_55_/GPa	C_66_/GPa
Cr_7_C_3_	459.9	262.7	251.0	512.6	280.2	525.1	186.2	142.9	114.2
	(410.1, *^c^* 409) *^a^*	(252, *^a^* 241.1) *^c^*	(227, *^a^* 203.7) *^c^*	(441, *^c^* 376) *^a^*	(333, *^a^* 257.3) *^c^*	(459.5, *^c^* 409) *^a^*	(168, *^c^* 145) *^a^*	(124.3, *^c^* 123) *^a^*	(108.3, *^c^* 82) *^a^*
Cr_3_C_2_	463.0	212.5	228.2	543.6	227.5	495.3	235.8	116.1	240.0
	(484, *^a^* 447.1) *^c^*	(229, *^a^* 217.5) *^c^*	(243.3, *^c^* 243) *^a^*	(554, *^a^* 545.3) *^c^*	(244, *^a^* 217.9) *^c^*	(491, *^a^* 471.2) *^c^*	(237.7, *^c^* 230) *^a^*	(116.6, *^c^* 111) *^a^*	(241.3, *^c^* 235) *^a^*
Cr_23_C_6_	486.3	201.0					149.6		
	(487, *^f^* 481, *^a^* 473.8) *^c^*	(209, *^a^* 200, *^f^* 186.6) *^c^*					(149, *^f^* 146.7, *^c^* 138) *^a^*		
Phase	C_11_/C_33_	C_12_/ C_13_	C_44_/C_66_	B/GPa	G/GPa	*B*/*G*	*E*/GPa	*v*	
Cr_7_C_3_	0.88	1.05	1.63	341.7	133.1	2.57	353.4	0.33	
				(312, *^a^* 311.7, *^e^* 309, *^b^* 300.6) *^c^*	(143.9, *^d^* 118.0, *^c^* 82) *^a^*	(2.55) *^a^*	(374, *^e^* 371, *^b^* 313.0, *^c^* 226) *^a^*	(0.38, *^a^* 0.33) *^c^*	
Cr_3_C_2_	0.94	0.93	0.98	314.7	166.1	1.89	423.7	0.28	
				(329, *^a^* 312.9) *^c^*	(162.1, *^c^* 162) *^a^*	(1.93) *^a^*	(416, *^a^* 414.7) *^c^*	(0.29, *^a^* 0.28) *^c^*	
Cr_23_C_6_	1.00	1.00	1.00	296.1	146.8	2.02	377.9	0.29	
				(300, *^a^* 282.3) *^c^*	(145.4, *^c^* 137) *^a^*	(1.94) *^a^*	(372.3, *^c^* 357) *^a^*	(0.30, *^a^* 0.28) *^c^*	

*^a^* Exp. data from H. Kleykamp [8]. *^b^* Cal. data from Music et al. [7]. *^c^* Cal. data from Min et al. [18]. *^d^* Cal. data from Price et al. [31]. *^e^* Cal. data from Xiao et al. [32]. *^f^* Exp. data from Henriksson et al. [33].

**Table 3:** Atomic orbit population of Cr_7_C_3_, Cr_3_C_2_ and Cr_23_C_6_ at zero pressure.

*Phase*	*Species*	*s*	*p*	*d*	*Total*	*Charge*(*e*)
Cr_7_C_3_	C	1.38	3.16		4.53	−0.53
	C	1.38	3.17		4.55	−0.55
	Cr	2.08	6.66	5.01	13.74	0.26
	Cr Cr	2.09 2.07	6.69 6.76	5.00 5.00	13.78 13.82	0.22 0.18
Cr_3_C_2_	C	1.40	3.09		4.49	−0.49
	C	1.40	3.11		4.51	−0.51
	Cr Cr Cr	2.13 2.14 2.15	6.61 6.54 6.50	4.96 4.99 4.98	13.70 13.67 13.63	0.30 0.33 0.37
Cr_23_C_6_	C Cr Cr	1.38 2.17 2.10	3.21 6.73 6.76	5.00 5.00	13.90 13.86	−0.59 0.10 0.14

**Table 4:** The volume of unit cell (Ω, ${Å}^{3}$) the *v* type bond density per ${Å}^{3}$($N_{b}^{\nu }$), the hardness of *u* type bond ($H_{\nu }^{u}$, GPa) and theoretical hardness (*H*, GPa) of Cr_7_C_3_, Cr_3_C_2_ and Cr_23_C_6_ at zero pressure.

Phase	Bond	$\Omega ({Å}^{3})$	$N_{b}^{\nu }\;$	$H_{\nu }^{u}/\mathrm{GPa}$	*H_Gao/_*GPa
Cr_7_C_3_	C-Cr	364.74	0.192	17.27	13.5 (18.3 *^a^*)
	C-Cr C-Cr C-Cr C-Cr C-Cr			17.19 15.55 14.81 14.50 13.67	
Cr_3_C_2_	C-Cr C-Cr C-Cr C-Cr C-Cr C-Cr	175.47	0.160	29.03 27.25 22.35 19.81 8.46 8.17	18.2 (20.9 *^a^*)

Cr_23_C_6_	C-Cr	1178.28	0.163	11.24	10.1 (13.2 *^a^*)
	C-Cr			9.07	

*^a^* Cal. data from Min et al. [18].

**Table 5:** The calculated anisotropic hardness of binary chromium carbides.

Phase	Chemical Bond	*H*_v*ani*_/GPa					*f_w_*	*H*_v_/ GPa
		[100]	[010]	[001]	[110]	[111]		
Cr_7_C_3_	Cr-C	24.93	24.16	21.05	**26.53**	25.79	0.019	26.17
Cr_3_C_2_	Cr-C	22.78	21.47	21.57	22.23	**24.16**	0.019	23.30
Cr_23_C_6_	Cr-C	16.42	15.18	**21.91**	17.86	21.15	0.041	19.09

**Table 6:** Theoretically calculated thermal properties of chromium carbides, including $v_{1}\left(m\cdot s^{-1}\right)$**,** $v_{s}\left(m\cdot s^{-1}\right)$, $v_{m}\left(m\cdot s^{-1}\right)$ and Θ*_D_*(K) at zero pressure.

Phase	$v_{1}$ $/m\cdot s^{-1}$	$v_{s}$ $/m\cdot s^{-1}$	$v_{m}$ $/m\cdot s^{-1}$	$\Theta_{D}/K$
${\mathrm{Cr}}_{7}C_{3}$	10,894.29(8051.65 *^a^*)	5516.13(4087.19 *^a^*)	6183.41(4580.90 *^a^*)	731(785 *^a^*, 646 *^b^*)
${\mathrm{Cr}}_{3}C_{2}$	11,447.63(8812.80 *^a^*)	6371.63(4878.25 *^a^*)	7095.38(5343.72 *^a^*)	850(785 *^a^*, 785 *^b^*)
${\mathrm{Cr}}_{23}C_{6}$	10,692.17(8152.31 *^a^*)	5841.449(4504.88 *^a^*)	6514.34(5019.43 *^a^*)	744(691 *^a^*, 671 *^b^*)

*^a^* Cal. data from Min et al. [18]. *^b^* Cal. data from Jang C et al. [9].

## Data Availability

Not applicable.
